# Correlations and forecast of death tolls in the Syrian conflict

**DOI:** 10.1038/s41598-017-15945-x

**Published:** 2017-11-16

**Authors:** Kazuki Fujita, Shigeru Shinomoto, Luis E. C. Rocha

**Affiliations:** 1 Department of Physics, Kyoto University, Kyoto, 606-8502 Japan; 2 Department of Sociology, Stockholm University, Universitetsvägen 10B, Stockholm, S-10691 Sweden; 3 Department of Public Health Sciences, Karolinska Institutet, 18A Tomtebodavägen, Stockholm, S-17177 Sweden

## Abstract

The Syrian armed conflict has been ongoing since 2011 and has already caused thousands of deaths. The analysis of death tolls helps to understand the dynamics of the conflict and to better allocate resources and aid to the affected areas. In this article, we use information on the daily number of deaths to study temporal and spatial correlations in the data, and exploit this information to forecast events of deaths. We found that the number of violent deaths per day in Syria varies more widely than that in England in which non-violent deaths dominate. We have identified strong positive auto-correlations in Syrian cities and non-trivial cross-correlations across some of them. The results indicate synchronization in the number of deaths at different times and locations, suggesting respectively that local attacks are followed by more attacks at subsequent days and that coordinated attacks may also take place across different locations. Thus the analysis of high temporal resolution data across multiple cities makes it possible to infer attack strategies, warn potential occurrence of future events, and hopefully avoid further deaths.

## Introduction

The outbreak of the current Syrian armed conflict occurred in March 2011 as a consequence of protests demanding democratic reforms and the end of the current government. These protests quickly escalated and within weeks were widespread in key cities all over Syria, eventually giving rise to groups against and in favor of the government^1^. Since then, the Syrian conflict has been marked by a large number of deaths of both civilians and military personnel. Although a matter of debate^2^, estimates claim that 470,000 people have been killed and at least 3 million refuged or migrated to foreign countries by the end of 2015^3^.

The dynamics of wars is complex and involves interdependent cultural, ethnic, political and economic variables that are difficult to model and predict^4–6^. Modeling efforts have been employed to forecast the outbreak of conflicts^6–11^ and to understand their dynamics^5,12–14^. There is also much interest on estimating the number of casualties and death tolls. Such information helps to allocate resources, estimate the magnitude of the conflict, develop war strategies from the military and political points of view^15^, and to quantify the burden of the war on health systems (needed for example to deliver humanitarian aid) and on the society^16–18^. Reliable data on death tolls are difficult to obtain and different methods exist to improve data collection during and after the conflict^17^. Higher resolution temporal data sets (at daily and weekly resolution) have however became increasingly available in recent years, allowing researchers to employ advanced methods of time-series analysis to make predictions on death tolls^19–22^, extreme massacres^23^ and to study the dynamics of conflicts^24–26^.

In this article, we study the daily time series of death tolls in the current Syrian conflict and look for the possibility of detecting signs of war-related tragic events based on temporal correlation within individual cities and spatial correlation across different cities. We compare these results to the statistics of a benchmark country, England, which is a representative country not undergoing domestic armed conflicts, in which the daily number of deaths at different cities is expected to have no direct causal relation. We exploit these correlations to improve the forecast of violent deaths in Syria, information that can be used to better allocate resources and aid to affected regions.

We firstly investigate auto-correlations in the number of deaths in individual cities and cross-correlations across cities, and find that these correlations are significant and positive in Syria, suggesting a possible coordination of events in multiple cities. Secondly, we perform simulations by assimilating models to the characteristics of the real data such as slow non-stationary fluctuations or rapid daily correlations within each city, and examine the extent to which the observed temporal and inter-city correlations are explained by these apparent characteristics. Thirdly, we carry out the Granger causality analysis^27,28^ to see if there are statistical causal relations across different cities. Finally, we attempt to predict the number of deaths; given a set of data for each country, we fix the parameters of prediction models using the initial part of the time series and then apply the models to the rest to validate if the models give a reasonable prediction on the future. The predictability depends not on the model but essentially on whether there is statistical causal relation between cities in each data set. We find for Syrian data that the vector auto-regression (VAR)^29^ model that takes account of inter-city correlations outperforms the auto-regression (AR)^29^ model that uses only single time series of individual cities separately. This indicates that the information of war-related events occurring in some cities may be used for warning of potential occurrence of future events in other cities.

## Results

### Death tolls

**Figure 1:**
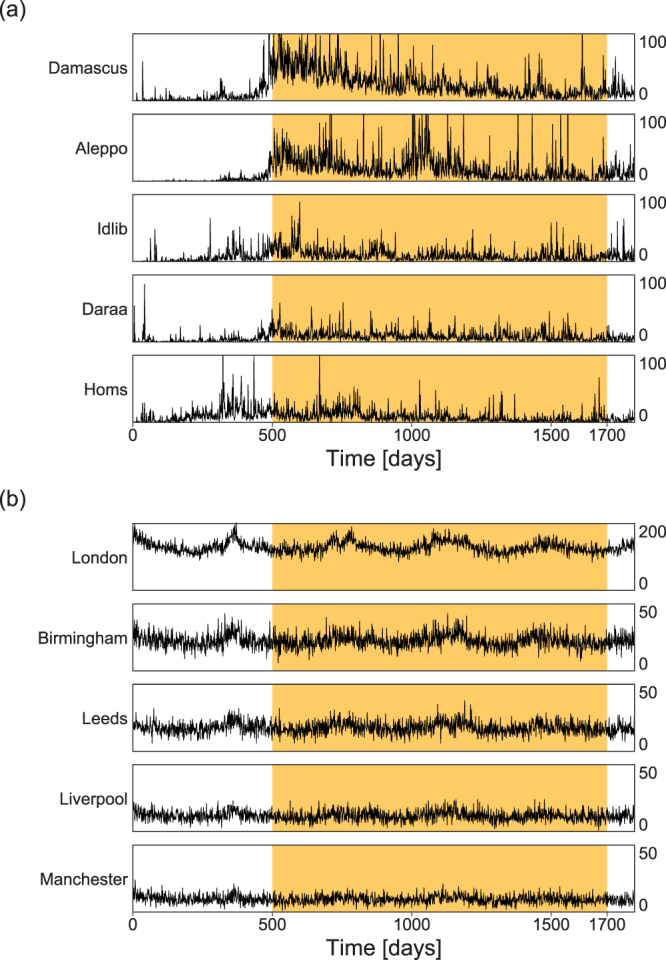
Daily time series of the number of deaths. The figure shows the number of deaths per day (y-axis) in different cities of (**a**) Syria and (**b**) England. The highlighted interval shows the time period of 1200 days for which we have performed the correlation and the Granger causality analyses. For the prediction analysis, the initial 600 days (500 to 1099 in Fig. 1(a)) is used to fix the parameters of the prediction models, and the remaining 600 days (from 1100 to 1699) is used to examine the predictability.

The data used in this study come from The Violations Documentation Center (VDC) in Syria (www.vdc-sy.info). All data analyzed during this study are included in the Supplementary Information files. The VDC has been collecting information on death tolls in the Syrian conflict since June 2011 and retrospectively since the outbreak of the conflict in March 18, 2011. We aggregate data at the province level, adding together adults, children, civilians and military personnel to get a unified number of deaths per day. Figure 1(a) shows the time-series of death tolls after the outbreak, as to the top 5 provinces with most casualties, i.e. Damascus (including the suburbs), Aleppo, Idlib, Daraa and Homs. We focus our analysis in the times after the shock, starting at 500 days from the outbreak of the conflict, and during the following 1200 days.

The office for National Statistics (www.ons.gov.uk) provides the daily number of deaths in England, that we use as a reference, from 2010 to 2014. We select 5 large cities in England: the greater London area (hereafter referred simply as London), Birmingham, Leeds, Liverpool and Manchester. We also aggregate adults and children in this case. Figure 1(b) shows a seasonal pattern in which deaths are more common during winter in all studied cities. We match the starting dates in the two datasets.

**Figure 2:**
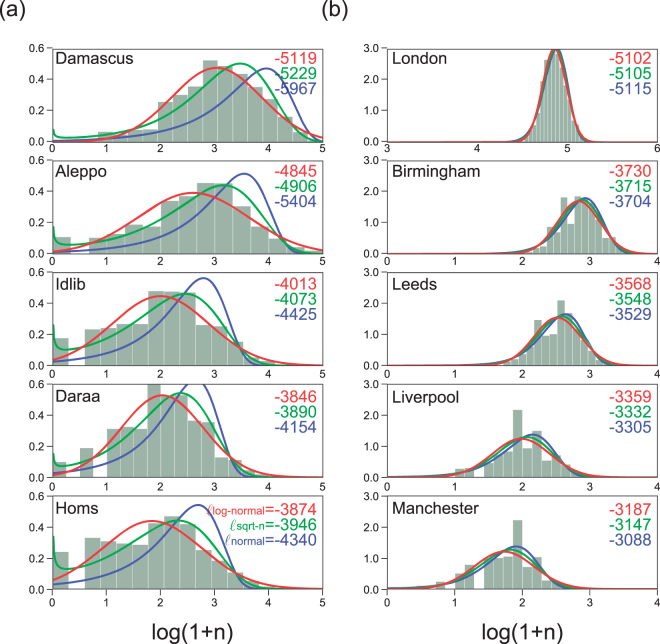
Distribution of number of deaths per day. (**a**) Syrian and (**b**) English cities. Distributions of $x=\,\mathrm{log}\,\mathrm{(1}+n)$ are plotted. The normal distributions of variables $x=n$, $x=\sqrt{n}$ and $x=\,\mathrm{log}\,\mathrm{(1}+n)$ fitted to each data are displayed on top of the histograms, with their goodness-of-fit represented in terms of the log-likelihood $\ell =\,\mathrm{log}\,L$. The bin-size of the histograms is determined by Scott’s rule that provides the best fitting for a compact set of data, so to minimize the mean square error^35^.

Figure 1 suggests large variation in the number of deaths in the case of Syria but not in the case of England. To study these fluctuations, we build a histogram of the number of deaths per day and analyze different parametric models to the data (Fig. 2). We have modeled three versions of the normal distribution of the transformed variables $x=n$, $x=\sqrt{n}$ and $x=\,\mathrm{log}\,\mathrm{(1}+n)$, where $n$ is the original number of deaths per day. The model goodness-of-fit was corroborated by estimating the log-likelihood $\ell \equiv \,\mathrm{log}\,L$ of each model to the data. Our analysis indicates that the log-normal distribution (i.e. normal with $x=\,\mathrm{log}(n+\mathrm{1)}$) is the best model (highest $\ell$) among the three alternatives for the Syrian data but all models give similar results for the English data.

The log-normal is a non-symmetric right skewed distribution in $n$, meaning that values much larger than the mean, i.e. many deaths in a single day, are relatively common in comparison to the standard normal distribution. Although alternative models could be tried, the log-normal distribution is appropriate and a standard choice when the underlying process consists of multiplicative random variables^30^. This is a reasonable assumption in our case given that the effects of shooting or bombing are non-linear and an attack may trigger multiplicative attacks locally or at different locations. Gomez-Lievano and collaborators^31^ also suggest that a log-normal distribution is an appropriate model of violent deaths (homicides) in the context of Brazil, Colombia and Mexico. In the literature, different mechanisms have been proposed to generate log-normal distribution of events^32^. Lewis and collaborators^21^, for example, recently suggested that a self-exciting point process may explain up to $\mathrm{50 \% }$ of the violent deaths in the case of Iraq between the years 2003 and 2007. It remains an open question to determine the specific mechanisms driving violent deaths in our context and further analysis is necessary.

The Syrian data only counts violent deaths. The English data counts both violent and non-violent deaths. A more detailed model for England could thus combine log-normal and normal distributions to account simultaneously to both violent and non-violent deaths. However, the majority (more than $\mathrm{90 \% }$) of deaths in England are non-violent (e.g. non-communicable diseases–www.ons.gov.uk) and a sum of independent Bernoulli random variables given rise to a normal distribution is a reasonable model. Though most English cities exhibited slightly larger likelihood for the normal distribution, Syrian data in question exhibited absolutely larger likelihood for the log-normal. Accordingly, from now on, we analyze all the time series (including English data) using the transformed variables $x=\,\mathrm{log}\,\mathrm{(1}+n)$. Note that the results are essentially unchanged even if we consider $x=n$ for modeling the English data.

### Correlation Analysis

The correlation analysis reveals the direction and strength of the relationship between two time-series, or of the same time-series at different times^29^. In our context, we will analyze the temporal structure of the daily deaths in individual cities (i.e. auto-correlation) and the spatial correlation across cities in the same country (i.e. cross-correlation). The cross-correlation of the daily deaths between cities $i$ and $j$ is given by

1 $${{\varphi }}_{ij}(t)=\frac{\frac{1}{T-t}\sum _{s=1}^{T-t}({x}_{i}(s+t)-{\bar{x}}_{i})({x}_{j}(s)-{\bar{x}}_{j})}{\frac{1}{T}{((\sum _{s=1}^{T}{({x}_{i}(s)-{\bar{x}}_{i})}^{2})(\sum _{s=1}^{T}{({x}_{j}(s)-{\bar{x}}_{j})}^{2}))}^{\frac{1}{2}}}$$

where $t$ is the time difference measured in the unit of day, $T=1200$ in our case, and ${\bar{x}}_{i}\equiv {\sum }_{t=1}^{T}\,{x}_{i}(t)/T$. The auto-correlation in city $i$ is thus given by ${{\varphi }}_{ii}(t)$. The value of ${{\varphi }}_{ij}(t)$ varies between $-1$ (negatively correlated) and $+1$ (positively correlated) at each time $t$, with ${{\varphi }}_{ij}(t)=0$ indicating no correlation.

**Figure 3:**
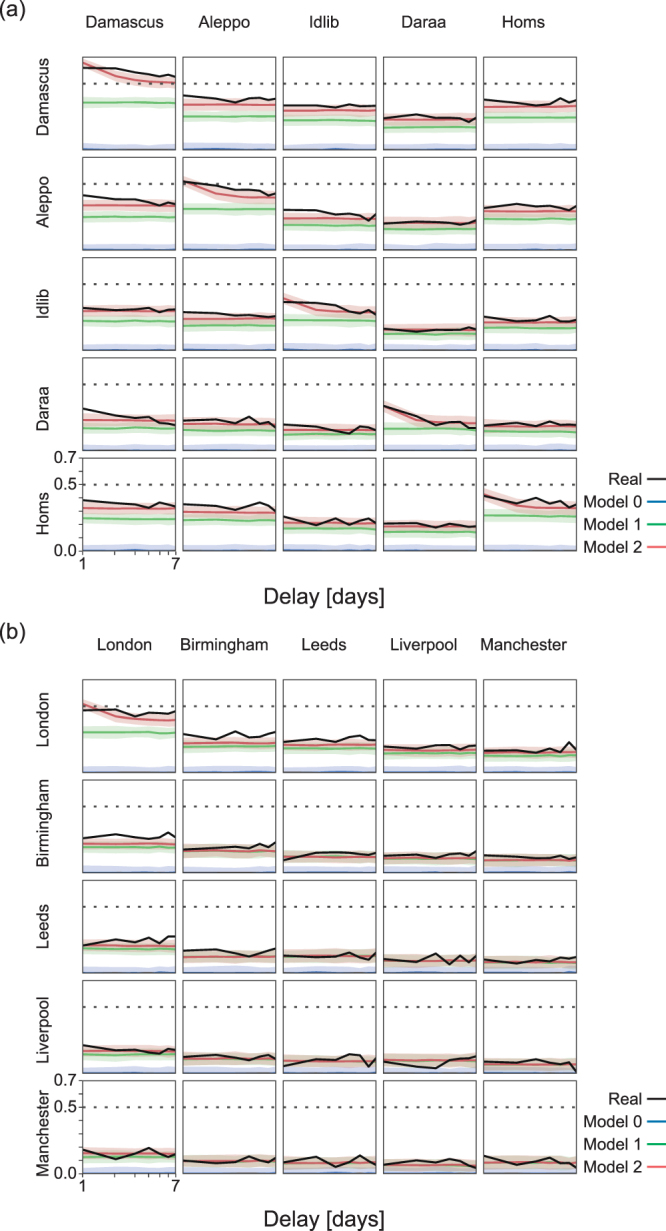
Correlation analysis. Temporal and spatial correlation represented by the auto- and cross-correlations across cities, $\{{{\varphi }}_{ij}(t)\}$ (y-axis) in (**a**) Syria and (**b**) England. The real data is show by black curves. Model 0 (blue curves) represents a stationary time series of independent Gaussian random numbers, given the mean and variance of each city. Model 1 (green curves) takes account of slow non-stationary modulation in each city. Model 2 (red curves) takes account of daily correlation. The memory parameters were chosen as ${h}_{{\rm{Damascus}}}=\mathrm{0.3,}\,{h}_{{\rm{Aleppo}}}=\mathrm{0.2,}\,{h}_{{\rm{Idlib}}}=\mathrm{0.15,}\,{h}_{{\rm{Daraa}}}=\mathrm{0.15,}\,{h}_{{\rm{Homs}}}=0.15$, ${h}_{{\rm{London}}}=\mathrm{0.2,}\,{h}_{{\rm{Birmingham}}}=\mathrm{0,}$ ${h}_{{\rm{Leeds}}}=\mathrm{0,}\,{h}_{{\rm{Liverpool}}}=\mathrm{0,}\,{h}_{{\rm{Manchester}}}=0$. The shaded area represents the $\mathrm{90 \% }$ interval (obtained by repeating simulations) of the stochastic deviation of models 0, 1, and 2. The delay $t$ in the x-axis is represented in the logarithmic scale. Dotted lines are used as eye-guides and indicate $\{{{\varphi }}_{ij}(t)\}=0.5$.

The correlation functions $\{{{\varphi }}_{ij}(t)\}$ computed for the time series of Syria and England are displayed in Fig. 3(a) and (b), respectively. The diagonal elements represent auto-correlations in individual cities, i.e. $\{{{\varphi }}_{ii}(t)\}$. For Syria, the prominent positive auto-correlation lasting for a week, particularly in Damascus (${{\varphi }}_{11}(t){\sim }0.6$) and Aleppo ($0.4\lesssim {{\varphi }}_{22}(t)\lesssim 0.5$) but also in Homs ($0.3\lesssim {{\varphi }}_{55}(t)\lesssim 0.4$), indicates that high (low) death tolls in one day are followed by high (low) death tolls on the next day in the same city. The correlation analysis does not necessarily mean causality but the results suggest that individual war-related events may have caused a number of deaths in the subsequent days or that major attacks (and thus deaths) trigger a series of new attacks with further deaths. Similarly, peaceful days are followed by further peaceful days. Note that auto-correlation is also present in London to some extent (${{\varphi }}_{11}(t)\sim 0.4$), where war-related conflicts are inexistent, but we shall see that this is mostly due to seasonal variation.

The spatial, inter-city or cross-correlation is represented as the off-diagonal elements in Fig. 3. We observe positive cross-correlation across Syrian cities, in particular from Damascus to Aleppo (${{\varphi }}_{21}(t) > 0.3$, from Damascus to Idlib (${{\varphi }}_{31}(t)\sim 0.3$), and from Damascus to Homs (${{\varphi }}_{51}(t) > 0.3$). These correlations are not as strong as the auto-correlations but their positive direction and strength suggest that high (small) death tolls in Damascus are followed by high (small) death tolls in Aleppo, Idlib and Homs (Fig. 3(a)). Similarly, significant cross-correlation is observed from Homs to Aleppo (${{\varphi }}_{25}(t) > 0.3$), from Aleppo, Idlib and Homs to Damascus, respectively ${{\varphi }}_{12}(t) > 0.3$, ${{\varphi }}_{13}(t)\sim 0.3$ and ${{\varphi }}_{15}(t) > 0.3$, and from Aleppo to Homs (${{\varphi }}_{52}(t) > 0.3$). Note that some cross-correlation is also present in a few cases in England, though at a relatively low intensity (Fig. 3(b)).

Figure 1 indicates that non-stationary fluctuations of long timescale are taking place commonly across different cities in each country; the death tolls in Syria have been slowly decreasing on average (Fig. 1(a)), while those in England exhibit seasonal modulation (Fig. 1(b)). To examine the extent to which the observed temporal and spatial correlations measured in the real data are explained by these slow fluctuations, we create three assimilated time series null models for each city and repeat the correlation analysis for each case. Each model reproduces different features of the time series, with increasing complexity.

#### Model 0: Stationary uncorrelated time series

We first generate a series of independent Gaussian random values ${\xi }_{i}(t)$, given the mean (${\bar{x}}_{i}$) and variance (${\bar{x}}_{i}^{2}-{\bar{x}}_{i}^{2}$) of each city $i$ (see Table 1):

2 $${x}_{i}^{\mathrm{(0)}}(t)={\xi }_{i}(t\mathrm{).}$$

**Table 1:** Mean (${\bar{x}}_{i}$) and variance (${\bar{x}}_{i}^{2}-{\bar{x}}_{i}^{2}$) of the number of deaths in Syrian and English cities. The number of deaths is measured as $x=\,\mathrm{log}\,\mathrm{(1}+n)$.

Syrian Cities	Mean	Variance
Damascus	3.03	0.83
Aleppo	2.60	1.01
Idlib	1.99	0.89
Daraa	2.03	0.75
Homs	1.84	0.91
**English Cities**	**Mean**	**Variance**
London	4.86	0.13
Birmingham	3.12	0.23
Leeds	2.89	0.26
Liverpool	2.48	0.32
Manchester	2.3	0.33

#### Model 1: Non-stationary uncorrelated time series

We modulate the stationary time series of Model 0 according to the slow modulation observed in each city, which can be obtained by smoothing the original data with the Gaussian kernel with the standard deviation of $k=10$ days,

3 $${\rm{\Delta }}{\tilde{x}}_{i}(t)=\sum _{s=1}^{T}\,{x}_{i}(s)\frac{1}{\sqrt{2\pi }k}{e}^{-\frac{{(t-s)}^{2}}{2{k}^{2}}}-{\bar{x}}_{i}\mathrm{.}$$

The smoothed modulation is added to the time series of Model 0 as

4 $${x}_{i}^{\mathrm{(1)}}(t)={x}_{i}^{\mathrm{(0)}}(t)+{\rm{\Delta }}{\tilde{x}}_{i}(t\mathrm{).}$$

This addition in terms of the logarithmic coefficient $x=\,\mathrm{log}\,\mathrm{(1}+n)$ corresponds to multiplying the modulation to the original number of deaths.

#### Model 2: Non-stationary correlated time series

The strong temporal correlation ${{\varphi }}_{ii}(t)$ lasting for a few days in Syrian cities may be reproduced by adding memory ${h}_{i}$ to the random variable ${y}_{i}(t)$, such that

5 $${y}_{i}(t)=((1-{h}_{i}){\xi }_{i}(t)+{h}_{i}{y}_{i}(t-1).$$

We might add higher order memory terms if needed. A stationary correlated time series may be constructed by iterating this equation. By adding the slow fluctuation ${\rm{\Delta }}{\tilde{x}}_{i}(t)$ (Eq. 3) to the stationary time series ${y}_{i}(t)$, we obtain a non-stationary correlated time series:

6 $${x}_{i}^{\mathrm{(2)}}(t)={y}_{i}(t)+{\rm{\Delta }}{\tilde{x}}_{i}(t\mathrm{).}$$

Figure 3 summarizes the results of the correlation analysis (Eq. 1) applied to Models 0, 1, and 2 in comparison to the real data. As expected, the uncorrelated stationary time series (Model 0) did not exhibit significant correlation. Model 1 that adopted the slow modulation has reproduced the most part of the slow correlations in English data, but has not succeeded in reproducing the strong auto-correlation and cross-correlation in Syrian data. Model 2 was able to reproduce the strong auto-correlation of the Syrian data by suitably accommodating the memory parameter ${h}_{i}$ (Fig. 3). Nevertheless, the strong correlations across real Syrian cities (and weaker correlations across real English cities) were not fully reproduced with Model 2. These results suggest that observed inter-city correlations are genuine and these violent death tolls are correlated between some cities. For example, an increase (decrease) of deaths in Damascus is accompanied by an increase (decrease) of deaths in Aleppo, Daraa and Homs, or an increase (decrease) in Aleppo is followed by an increase (decrease) in Homs. The weak cross-correlations observed in some English cities may reflect the known effect of synchronization of deaths due to seasonality with peaks during winter months^33^. The large number of deaths and seasonality may also explain the larger auto-correlations observed in London but not in other English cities.

Altogether, these results suggest that there may be some coordination in the attacks (and consequently deaths) at the different Syrian cities, or in other words, attacks may spread to different locations. Although this effect is not as strong, it is significantly higher than one would expect if no violent conflict was going on across the country, given that cross-correlation is close to zero between cities in England. In the next section, we will look more carefully to these inter-city correlations and exploit this information to make better forecast of death tolls.

### Granger causality

We try to detect statistical causal relation between the different cities in both countries, using a standard pairwise-conditional Granger causality (GC) analysis^27,28^. The GC analysis indicates if the evolution of the number of deaths at city $i$ is better predicted by combining information from the past deaths in both cities $i$ and $j$ than if using only the past information of deaths in city $i$. This is an extension of the previous analysis because it measures the improvement on forecast in the presence of information from other variables.

**Figure 4:**
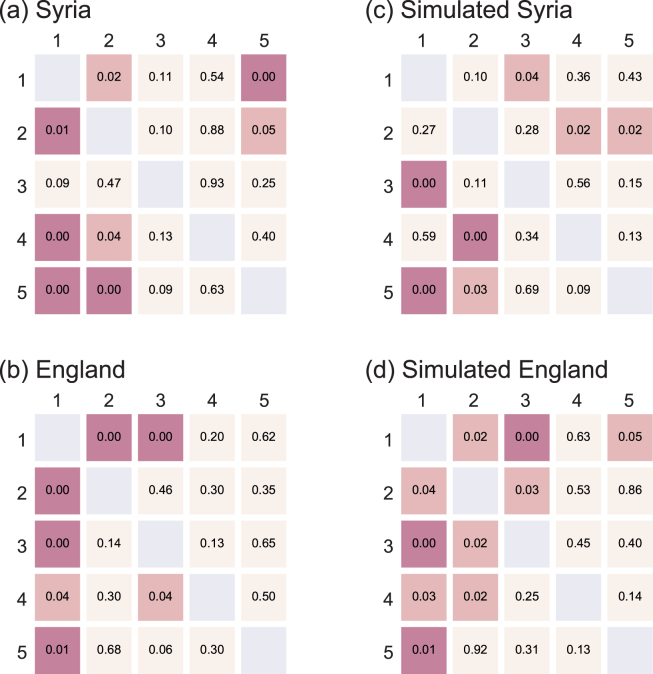
Granger causality analysis applied to the original data. The $p$-values of Granger causality for real (**a**) Syrian and (**b**) English cities, and for the simulated (**c**) Syrian and (**d**) English cities given by Model 2. For Syrian data: 1: Damascus; 2: Aleppo; 3: Idlib; 4: Daraa; 5: Homs. For English data: 1: London; 2: Birmingham; 3: Leeds; 4: Liverpool; 5: Manchester. Low $p$-values indicate statistical causality.

The naive GC analysis indicated many inter-city correlations (indicated by low $p$-values) not only in Syria (Fig. 4(a)) but also in England (Fig. 4(b)), although the direct causal relations between English cities are expected to be absent. The GC analysis is known to be vulnerable to non-stationary fluctuations and likely to suggest spurious correlations^34^. By applying the same GC analysis to the assimilated data (Model 2 described in the previous section), we also obtained similar correlations (Fig. 4(c) and (d)). The result suggests that the seasonal modulation in the number of deaths has induced the observed causality in the English cities.

The influence of slow non-stationary fluctuations (such as seasonality) to the GC analysis may be mitigated by analyzing the temporal difference^29^,

7 $${z}_{i}(t)\equiv {x}_{i}(t)-{x}_{i}(t-\mathrm{1).}$$

**Figure 5:**
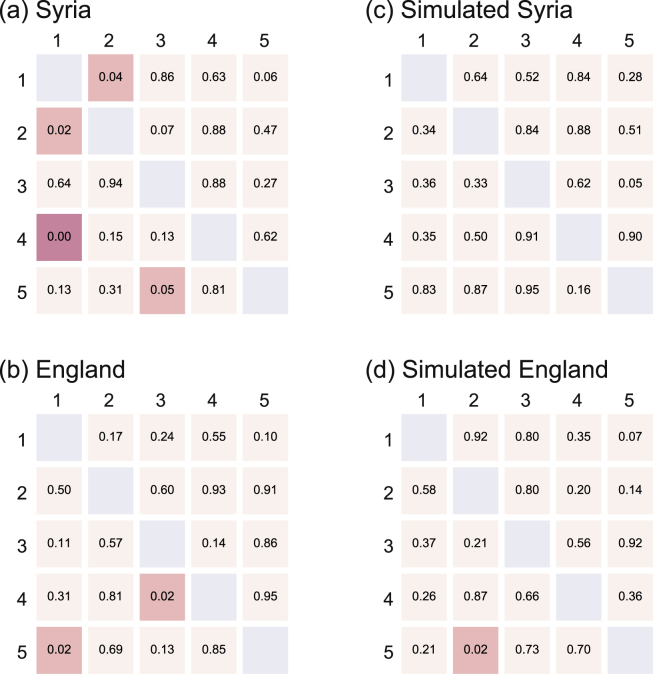
Granger causality analysis applied to the time difference data ${z}_{i}(t)\equiv {x}_{i}(t)-{x}_{i}(t-\mathrm{1)}$. The $p$-values of Granger causality for real (**a**) Syrian and (**b**) English cities, and for simulated (**c**) Syrian and (**d**) English cities given by Model 2. For Syrian data: 1: Damascus; 2: Aleppo; 3: Idlib; 4: Daraa; 5: Homs. For English data: 1: London; 2: Birmingham; 3: Leeds; 4: Liverpool; 5: Manchester. Low $p$-values indicate statistical causality.

In our case, this operation succeeded in removing inter-city correlations from not only the simulated Model 2 (Fig. 5(c) and (d)), but also from real English data (Fig. 5(b)). However, the real Syrian data is left with directed inter-city correlations from Damascus to Aleppo and Daraa, from Aleppo to Damascus, and from Idlib to Homs (low $p$-values in Fig. 5(a)). Thus the GC analysis indicates that death tolls are dependent across these Syrian cities but not across English or on simulated cities, in which the original causal relations were an effect of the slow fluctuations.

We note that “slow” and “fast” fluctuations should be treated with care. It is easier for the GC or other analysis methods to detect rapid changes in the number of events (in our case, death tolls). For example, the GC analysis may interpret “causal” if a deadly infectious disease propagates from city to city in a few days, even if there is no physical causal influence between cities. This is unavoidable, because the analysis method simply count the death tolls without inspecting the other information. A slower propagation of an infectious deadly disease such as the plague in the 14th century might also be detected using GC if the changes in the death tolls were large.

### Forecast

We attempt to predict the death tolls in Syria and England using the auto-regression (AR) model (Eq. 8) and the vector auto-regression (VAR) model (Eq. 9)^29^. We use the initial 600 days of the time series (500 to 1099 in Fig. 1(a)) to fix the model parameters, and apply the models to the remaining 600 days (from 1100 to 1699) to see if they give efficient prediction on the future number of deaths in each country.

The AR model only uses information of a single time series to forecast values of the corresponding time series, as given by

8 $${\hat{x}}_{i}(t)=c+\sum _{s=1}^{m}{a}_{i}(s){x}_{i}(t-s)+\varepsilon (t),$$

where ${\hat{x}}_{i}(t)$ represents the predicted value, ${\{{a}_{i}(s)\}}_{s=\mathrm{1,}\cdots ,m}$ are parameters determined with the temporal auto-correlation ${\{{{\varphi }}_{ii}(s)\}}_{i}$ (Eq. 1) computed for the initial 600 days of the time series, and $m$ is the order of regression. We take $m=5$ for the current analysis.

The VAR model uses the time series of all cities simultaneously to forecast values of all cities at once, as given by

9 $$\hat{x}(t)=c+\sum _{s\mathrm{=1}}^{m}{\bf{A}}(s)x(t-s)+\varepsilon (t),$$

where ${\bf{x}}(t)$ represents a vector comprising of five cities ${({x}_{1}(t),{x}_{2}(t),\cdots ,{x}_{5}(t))}^{t}$, and ${\bf{A}}(s)$ is a matrix whose elements are determined with the temporal correlations ${\{{{\varphi }}_{ij}(s)\}}_{ij}$ (Eq. 1).

If there are significant correlations between cities, there is room for the VAR model to make the better forecast in comparison to the AR model that only uses information of a single time series (i.e. single city). As a reference, these two models are compared with two simpler models: (i) predicting the future deaths simply with the number of deaths of the preceding date (Preceding), and (ii) predicting deaths with a single fixed value given by averaging over the number of deaths during the past first half of the time series (Average).

Table 2 demonstrates the performance of these four models for the Syrian and English cities in terms of the average prediction error $MSE\equiv {\sum }_{t=T+1}^{2T}{({\hat{x}}_{i}(t)-{x}_{i}(t))}^{2}/T$ over the testing period of $T=600$ days, where the forecast provided by each model is given by ${\hat{x}}_{i}(t)$ and the true value is given by ${x}_{i}(t)=\,\mathrm{log}(1+{n}_{i}(t))$. The AR and VAR models generally perform much better than the other two methods. Among the two regression models, VAR outperforms AR model for the Syrian data. The increase in the performance for the prediction errors ${\rm{\Delta }}\equiv (MS{E}_{{\rm{AR}}}-MS{E}_{{\rm{VAR}}})/MS{E}_{{\rm{AR}}}$ in Table 2 is large for three cities in Syria, in contrast to the negligible improvement in the English cities (less than $\mathrm{2 \% }$).

**Table 2:** Forecast error for Syrian and English cities. The table shows the mean square error ($MSE$) between the forecast provided by each model ${\hat{x}}_{i}(t)$ and the true value ${x}_{i}(t)$. The column ${\rm{\Delta }}$ stands for the improvement of the prediction errors of the VAR model in respect to the AR model.

	Preceding	Average	AR	VAR	${\rm{\Delta }}$($\%$)
Damascus	0.717	0.552	0.500	0.478	$+4.4$
Aleppo	1.383	1.064	1.016	1.006	$+1.0$
Idlib	1.295	0.823	0.787	0.791	$-0.5$
Daraa	0.901	0.638	0.635	0.570	$+10.2$
Homs	1.173	0.711	0.831	0.732	$+11.9$
London	0.017	0.017	0.011	0.011	$0$
Birmingham	0.088	0.059	0.052	0.051	$+1.9$
Leeds	0.110	0.066	0.061	0.060	$+1.6$
Liverpool	0.187	0.101	0.101	0.100	$+1.0$
Manchester	0.194	0.113	0.108	0.108	$0$

**Figure 6:**
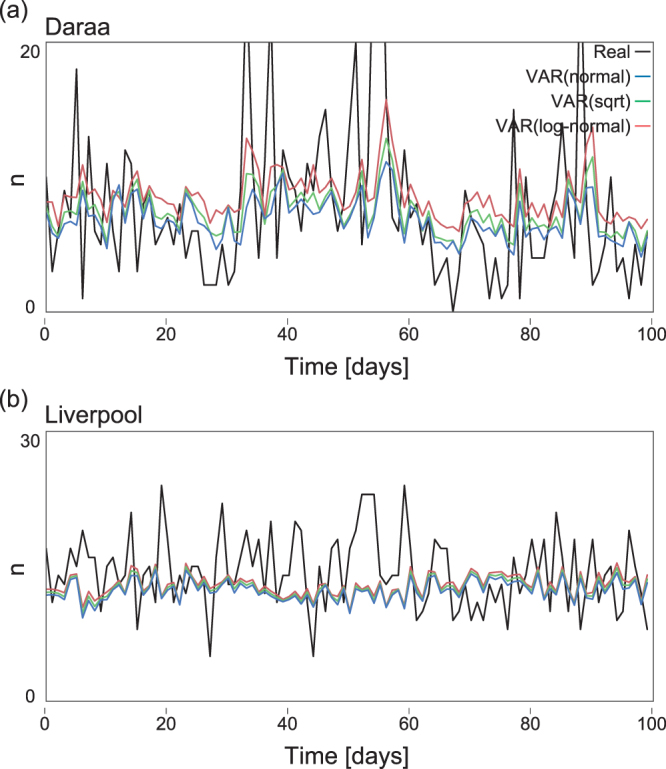
Prediction of the number of deaths. The plots show the number of deaths ($n$) from 1100 to 1199 days (i.e. the initial 100 days of the latter half of the time series) which is used for evaluating the predictability power of the models. (**a**) Syrian city of Daara and (**b**) English city of Liverpool. Predictions made by VAR of $x=n$, $x=\sqrt{n}$ and $x=\,\mathrm{log}\,\mathrm{(1}+n)$ are plotted on top of the real time series.

The comparison of prediction models in Table 2 is made assuming the log-normal distribution, or by setting $x=\,\mathrm{log}\,\mathrm{(1}+n)$. We firstly compared different kinds of distribution such as the normal distributions of variables $x=n$, $x=\sqrt{n}$ and $x=\,\mathrm{log}\,\mathrm{(1}+n)$ in terms of the likelihood values. Here we also compare them in terms of the performance of predicting the number of deaths. Figure 6(a) and (b) demonstrate the predictions made for the Syrian city of Daraa and the English city of Liverpool using the VAR of $x=n$, $x=\sqrt{n}$ and $x=\,\mathrm{log}\,\mathrm{(1}+n)$. It is observed that $x=\,\mathrm{log}\,\mathrm{(1}+n)$ is superior to $x=\sqrt{n}$ and $x=n$ for the data of Daraa, while three variables make no significant difference for the data of Liverpool.

**Figure 7:**
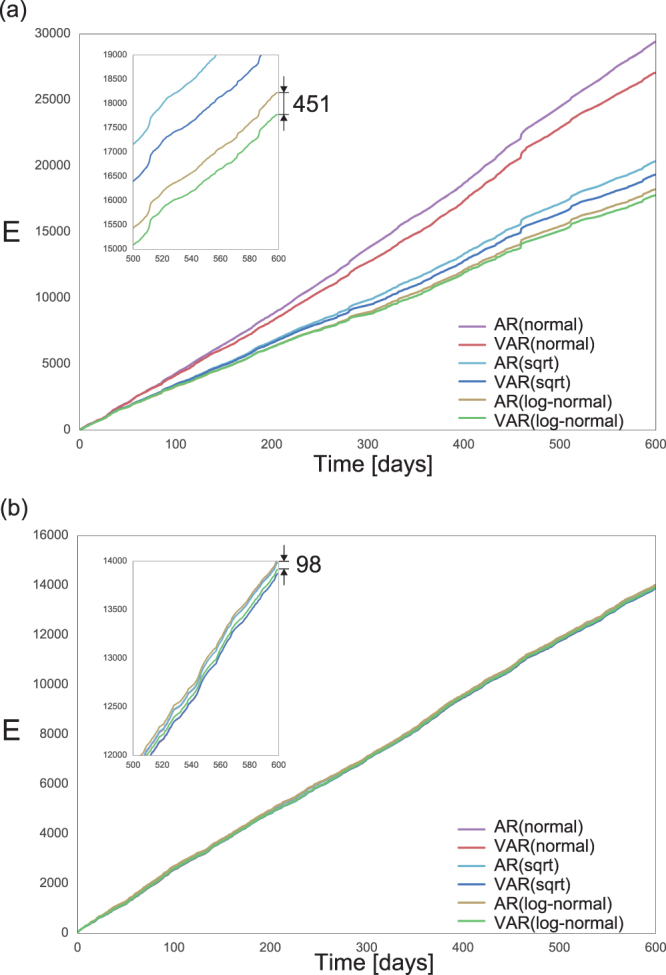
Prediction of the number of deaths. The plots show the cumulative difference $E$ (Eq. 10) between the predicted and the actual number of deaths from 1100 to 1699 days for the AR and VAR models, using three different transformed variables for (**a**) Syria and (**b**) England.

To better visualize the impact of these errors in the forecast of death tolls, we measure the total prediction error ($E$) in the unit of the number of dead people. Considering all five cities of each country together, we have

10 $$E=\sum _{i=1}^{5}\sum _{t=1}^{T}|{n}_{i}(t+T)-{\hat{n}}_{i}(t+T)|,$$

where $T=600$ days, ${n}_{i}(t)$ is the number of deaths of city $i$ at day $t$, and ${\hat{n}}_{i}(t)$ is the predicted value given by ${\hat{n}}_{i}(t)={x}_{i}(t)$ if we use the transformed variable ${x}_{i}(t)={n}_{i}(t)$, ${\hat{n}}_{i}(t)={\hat{x}}_{i}{(t)}^{2}$ if we use ${x}_{i}(t)=\sqrt{{n}_{i}(t)}$ and ${\hat{n}}_{i}(t)={e}^{{\hat{x}}_{i}(t)}-1$ if we use ${x}_{i}(t)=\,\mathrm{log}(1+{n}_{i}(t))$. Figure 7 shows that the VAR model using the transformed variable ${x}_{i}(t)=\,\mathrm{log}(1+{n}_{i}(t))$ gives the best prediction of future deaths. In the best case, the difference in the total prediction error between AR and VAR models corresponds to 451 people in the period of 600 days. In fact, for all transformed variables, the VAR model gives better prediction than the AR model, that is, a difference of 1010 deaths for ${x}_{i}(t)=\sqrt{{n}_{i}(t)}$ and 2337 deaths for ${x}_{i}(t)={n}_{i}(t)$. These differences between VAR and AR are substantially smaller in the case of England; the cumulative difference sums up to 98 deaths for ${x}_{i}(t)=\,\mathrm{log}(1+{n}_{i}(t))$. For the remaining, 115 deaths for ${x}_{i}(t)=\sqrt{{n}_{i}(t)}$ and 111 for ${x}_{i}(t)={n}_{i}(t)$.

These results indicate a significant improvement in the prediction power of death tolls in Syria when using information from multiple cities and the log-transformed variables. In comparison, the second best model (AR with log-transformed variables) predicts about 0.75 more deaths per day. While this might sound a small difference, it accumulates to about 23 people within a month. From the analytical point of view, these results emphasize that the cross-correlations in the death tolls at different cities in Syria are significantly higher than those in the English cities, in which all tested models give relatively similar results.

## Discussions

Armed conflicts typically cause significant life losses on all sides. Estimates of death tolls are important in order to quantify the magnitude of the war, encourage peacemaking and to allocate resources and humanitarian aid. The availability of high-resolution spatiotemporal data on the number of deaths allows researchers to analyze correlations between different cities at different times and to identify trends that could possibly be used to reduce future causalities.

In this paper, we use daily information on the number of deaths in a given city to study spatial and temporal correlations of death tolls in the current Syrian conflict and compared the results with the daily number of deaths in English cities that are not undergoing any domestic conflict. We have explored different models to remove potential virtual correlations in the empirical data, as was the case in English cities, mainly due to seasonality. Our analysis showed that significant positive auto-correlation exist in Syrian cities, meaning that days with a high (low) number of deaths in a particular city were followed by days with many (few) deaths in the same city, possibly reflecting a sequence of attacks within short periods triggered by a single attack. Similarly, we have also observed significant cross-correlation (i.e. spatial correlation) between some cities in Syria. This means that deaths in one city were accompanied by deaths at another city, for example, from Damascus to Aleppo and vice-versa, from Damascus to Daraa, and from Idlib to Homs. Given the available data, we cannot infer mechanistic causality in deaths between different cities but the cross-correlations and Granger causality analysis suggest that events are not completely random but coordinated attacks at multiple locations possibly take place. At the same time, correlations are typically not super strong and are not observed between all cities too. Such results are useful since one can exploit them to develop a warning system monitoring the sequence of events taking place at different days and cities aiming to better understand attack strategies and avoid further deaths. For example, since the number of deaths increases in both Damascus and Aleppo, attacks in one city may trigger warnings to the other.

We have also explored the possibilities to forecast death tolls at different cities. Our analysis have shown that due to the significant cross-correlations, improved forecast is obtained for Syria if using information from all cities simultaneously in a vector auto-regression model in comparison to single cities in independent auto-regression models. Contrastingly, the difference is very small (typically less than $\mathrm{2 \% }$) for the case of England. The important conclusion here is that death tolls during the conflict in Syria can be better predicted if information from multiple locations and times are simultaneously available. Such prediction could be used to organize the allocation of resources and aid during the conflict.

We observe that daily death tolls in Syria can be well-described by log-normal distributions which is in contrast to English cities, in which death tolls can be described equally well by the three distributions examined. This means that death events in Syria are not uniform and days with a much larger number of deaths than the average are expected during the conflict. This is consistent with the previous studies suggesting that the distribution of the number of violent deaths may be described by log-normal distributions given the multiplicative nature of violent events.

We should keep in mind that the numbers of casualties in Syria have been very large and the counting of deaths is a very difficult task. Accordingly the counts might have been accompanied with errors that could have affected the correlations between specific cities and the overall forecast exercise. Future work should focus on the analysis of different datasets to determine the intensity of the correlations and thus the possibilities for forecast on other contexts. Furthermore, it remains an open question to determine the mechanisms driving the positive correlations between the death tolls in different cities and in the same city at different times.

## Electronic supplementary material

Dataset 1

Dataset 2

Dataset 3

Dataset 4

Dataset 5

Dataset 6

Dataset 7

Dataset 8

Dataset 9

Dataset 10

Dataset 11
